# Enhancing Tensile Performance of Cemented Tailings Backfill Through 3D-Printed Polymer Lattices: Mechanical Properties and Microstructural Investigation

**DOI:** 10.3390/ma18143314

**Published:** 2025-07-14

**Authors:** Junzhou Huang, Lan Deng, Haotian Gao, Cai Wu, Juan Li, Daopei Zhu

**Affiliations:** 1 School of Civil Engineering, Hubei Engineering University, Xiaogan 432000, China; hjz13507290833@163.com; 2 Hubei 9D Mapping and Design Co., Ltd., Xiaogan 432000, China; 13971090855@163.com; 3 School of Software Engineering, Jiangxi University of Science and Technology, Nanchang 330013, China; 19863087208@163.com; 4 Fraunhofer-Institut für Holzforschung, Wilhelm-Klauditz-Institut, WKI. Riedenkamp 3, 38108 Braunschweig, Germany; juan.li@wki.fraunhofer.de; 5 Yichun Lithium New Energy Industry Research Institute, Jiangxi University of Science and Technology, Yichun 336023, China

**Keywords:** cemented tailings backfill, 3D-printed polymer lattice, mechanical properties, failure mode, DIC analysis

## Abstract

This study presents an innovative solution to improve the mechanical performance of traditional cemented tailings backfill (CTB) by incorporating 3D-printed polymer lattice (3DPPL) reinforcements. We systematically investigated three distinct 3DPPL configurations (four-column FC, six-column SC, and cross-shaped CO) through comprehensive experimental methods including Brazilian splitting tests, digital image correlation (DIC), and scanning electron microscopy (SEM). The results show that the 3DPPL reinforcement significantly enhances the CTB’s tensile properties, with the CO structure demonstrating the most substantial improvement—increasing the tensile strength by 85.6% (to 0.386 MPa) at a cement-to-tailings ratio of 1:8. The 3DPPL-modified CTB exhibited superior ductility and progressive failure characteristics, as evidenced by multi-stage load-deflection behavior and a significantly higher strain capacity (41.698–51.765%) compared to unreinforced specimens (2.504–4.841%). The reinforcement mechanism involved synergistic effects of macroscopic truss behavior and microscopic interfacial bonding, which effectively redistributed the stress and dissipated energy. This multi-scale approach successfully transforms CTB’s failure mode from brittle to progressive while optimizing both strength and toughness, providing a promising advancement for mine backfill material design.

## 1. Introduction

Extensive mining operations have led to severe ecological degradation, including soil erosion, groundwater contamination, and heightened geotechnical risks [1,2,3]. A major environmental concern is the long-term impact of mining waste, particularly heavy metal-laden tailings, which pose risks of ecosystem pollution through leaching and wind dispersion [4,5,6]. To enhance tailings utilization efficiency and develop safer and more stable mining practices, many operations now employ cemented tailings backfill (CTB) for tailings disposal [7,8,9]. CTB involves mixing tailings with cementitious binders and water before placement into underground stopes, offering a safe, cost-effective, and environmentally sustainable solution for modern mining [10,11,12]. However, conventional CTB suffers from poor mechanical properties, including low strength and susceptibility to failure under external loads [13,14]. It also exhibits rapid post-peak strength degradation and pronounced brittle failure behavior [15,16], raising safety concerns in mining operations [17]. Consequently, enhancing the mechanical performance of CTB is critically important [18,19,20]. Research indicates that the CTB strength is influenced by factors such as the cement-to-tailings ratio [21,22], solid content [23], and curing time [24,25].

To improve the performance of backfill materials, researchers have extensively studied the properties of cemented tailings backfill (CTB) by incorporating additives such as fly ash [26], fibers and cellulose [27,28,29], and waste rock [30,31]. These additives have been shown to enhance the mechanical properties of cementitious composites, including strength and toughness [32,33,34]. For instance, Su et al. [35] investigated the effects of polypropylene fiber length and strain rate on the dynamic mechanical properties of backfill, demonstrating that fibers significantly improve strength and toughness, with the dynamic compressive strength increasing with the strain rate. Zou et al. [36] optimized hybrid polypropylene and basalt fiber dosages, finding that 0.3% of each fiber provided the greatest mechanical improvement while mitigating damage. Similarly, Yin et al. [37] reported that polypropylene fibers enhance peak stress and crack resistance in sulfur tailings backfill, with an optimal content of 0.6%. Wang et al. [38] observed that basalt fibers improve CTB ductility and macro-mechanical performance, with 12 mm fibers at 1.5% content yielding the best results. Despite these benefits, fiber-reinforced backfill faces practical challenges, including pipeline clogging and uncertain long-term performance [39,40,41], prompting exploration of alternative materials [42,43,44]. Zhang et al. [45] found that increasing coal gangue and fly ash content reduces the unconfined compressive strength (UCS) and elastic modulus, with tensile failure dominating. Conversely, Jiang et al. [46] demonstrated that fly ash and blast furnace slag partially replacing cement enhanced the strength and durability of iron ore tailings. Cheng et al. [47] noted that substituting 30–50% cement with fly ash increased the UCS while reducing the porosity by 4%. Wang et al. [48] reported that nanocellulose materials initially raised the UCS before declining at higher concentrations. Cao et al. [49] showed that waste tire steel fibers (WTSF) improved ductility and damage tolerance despite lowering the UCS. Zhu et al. [50] and Wang et al. [51] highlighted waste rock’s role in strengthening cemented tailings backfill, with optimal performance at a gradation coefficient (*n* = 0.5) and 30% waste rock content. Similarly, Wu et al. [52] found that graded waste rock particles enhanced the compressive strength and ultrasonic pulse velocity by optimizing the internal structure.

Three-dimensional printing technology, a digital manufacturing method that builds three-dimensional objects through layer-by-layer material deposition, offers unique advantages for creating complex geometries while minimizing material waste [53]. Its applications have expanded across various fields, including healthcare [54], industrial manufacturing [55], textiles [56], and construction engineering [57]. Recently, researchers have begun investigating its potential for mine backfill applications. The technology’s inherent compatibility with lattice structure design makes it particularly suitable for fabricating such configurations [58,59,60]. Studies have demonstrated the effectiveness of 3D-printed polymer (3D-PP) structures in enhancing cemented tailings backfill (CTB) performance. Zou et al. [61] developed two 3D-PP models for CTB reinforcement, with three-point bending tests revealing significant improvements in flexural performance, particularly in bending strength and deflection capacity. Yaya et al. [62] found that 3D-PP geometry substantially increased the material toughness, inducing semi-brittle failure behavior. Zhang et al. [63] employed Brazilian splitting tests on CTB reinforced with three different 3D-printed polymer lattice (3D-PPL) configurations, revealing the micro-damage mechanisms and fracture evolution. Li et al. [64] demonstrated that 3D-PPL incorporation in cement-based composites not only enhanced the strength and ductility but also transformed the failure modes from single-crack to complex multiple-crack patterns involving both tensile and shear failures. Qin et al. [65,66] systematically evaluated various 3DPPL-reinforced CTB/CBC systems, consistently observing improved strength, toughness, and modified failure characteristics compared to conventional materials. These collective findings highlight how 3D-printed polymer structures can optimize mine backfill performance through strategic structural design.

The present study advances current knowledge by developing a hierarchical “column + cross” 3D-printed polymer lattice (3DPPL) architecture that uniquely combines macroscopic truss effects with microscopic interfacial interlocking. Unlike conventional fiber-reinforced or homogeneous foam-modified backfills, our approach introduces three innovative lattice geometries (FC, SC, and CO) specifically engineered for multi-scale stress redistribution in cemented tailings. The cross-shaped (CO) configuration represents a particular breakthrough, achieving an unprecedented 85.6% tensile strength improvement at a low cement content (1:8 ratio) while simultaneously transforming failure modes from brittle to progressive—a dual enhancement not reported in prior studies. Through systematic comparison of columnar versus biaxial reinforcement patterns, we establish quantitative structure–performance relationships using advanced DIC strain mapping and SEM interface analysis, revealing how lattice geometry governs crack deflection pathways and energy dissipation mechanisms. Furthermore, this work introduces novel environmental application parameters by demonstrating effective cement reduction (up to 50%) without compromising mechanical performance, addressing both sustainability and cost concerns in mine backfill operations. The combination of these technical innovations—unique lattice designs, comprehensive multi-scale characterization, and demonstrated environmental benefits—distinguishes this study from previous incremental improvements in backfill reinforcement and establishes a new framework for the performance-driven design of 3D-printed structures in geotechnical applications.

## 2. Test Raw Materials and Test Methods

### 2.1. Test Raw Materials

#### 2.1.1. Tailings and Cementitious Materials

**Figure 1:**
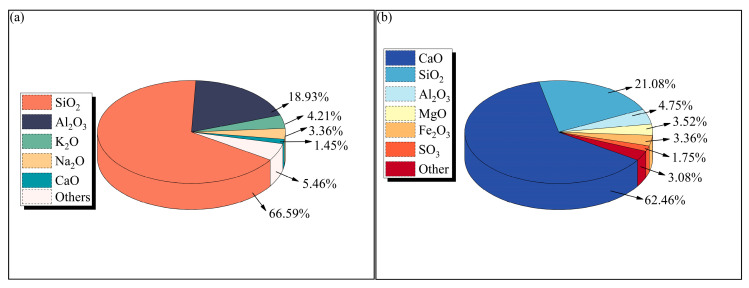
A pie chart of the oxide composition of the materials: (**a**) tailings; (**b**) cement.

**Figure 2:**
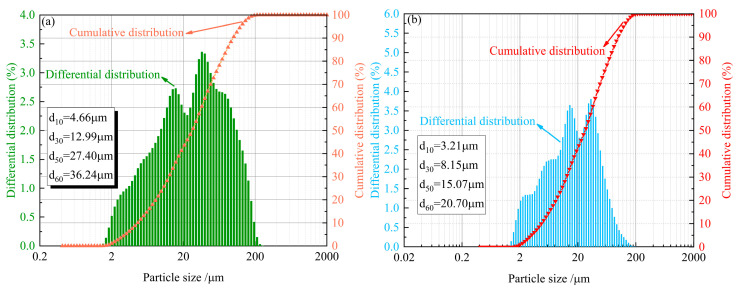
The PSD curve of the materials: (**a**) tailings; (**b**) cement.

The study utilized tailings sourced from a mineral processing plant in Yichun, Jiangxi Province, China. The tailings were dried and packaged in plastic bags to prevent moisture reabsorption prior to transportation. Ordinary Portland cement, the most commonly used binder in mining applications, was procured directly from a manufacturer, which come from Nanchang, Jiangxi Province, China. X-ray fluorescence (XRF) spectroscopy analysis determined the oxide composition of both materials (Figure 1), classifying the tailings as acidic according to established criteria [67]. The tailings contained 66.59% SiO_2_ and 18.93% Al_2_O_3_ as primary components, while the cement consisted mainly of 62.46% CaO and 21.08% SiO_2_. Laser granulometry analysis of the particle size distribution (PSD) (Figure 2) revealed both materials to be well-graded [68], with the tailings showing curvature and uniformity coefficients of 1.00 and 7.78, respectively, compared to 1.00 and 6.45 for cement.

#### 2.1.2. The Properties of 3D-Printed Polymers

**Figure 3:**
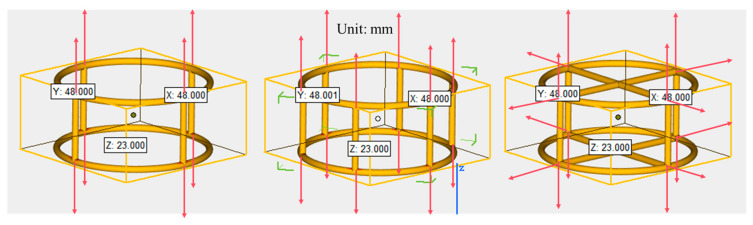
The model diagram of 3DPPL.

Building on our previous findings demonstrating white resin’s superior strength enhancement for CTB compared to black nylon and transparent resin [69], this study investigates the mechanical properties of CTB reinforced with white resin-based 3D-printed polymer lattices (3DPPL). Table 1 summarizes the 3DPPL’s physical and mechanical properties (as provided by the manufacturer). Three distinct cylindrical configurations were designed: four-column (FC), six-column (SC), and cross-shaped (CO) geometries (Figure 3). The 3D models (dimensions in mm) were created using Magics 2025 software and printed using an 800 Jinshi 3D SLA printer with the following parameters: 0.1 mm layer resolution, 0.2 mm layer height, 0.4 mm nozzle diameter, and 50 mm/s printing speed. Post-processing included isopropanol cleaning, UV curing, and support removal. All 3DPPL specimens featured 2 mm strut diameters with final dimensions of 23 mm height × 48 mm diameter—slightly smaller than the standard mold size (25 × 50 mm) to ensure proper placement during casting. The 3D printer and resin materials are from Shenzhen, Guangdong Province, China.

### 2.2. Test Method

#### The CTB Preparation Process with 3DPPL Added

**Figure 4:**
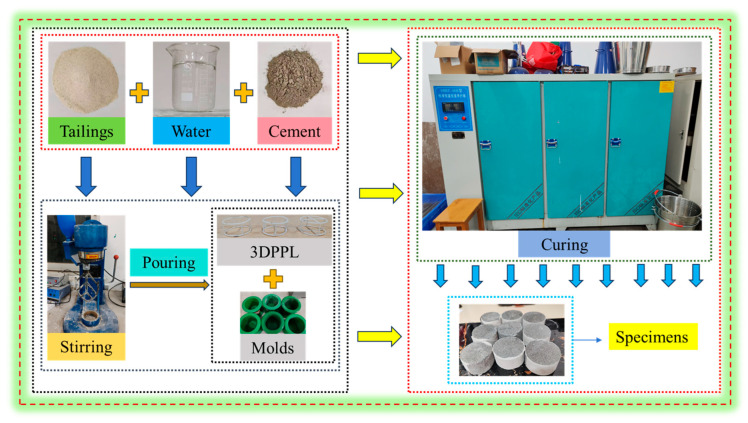
The preparation process of 3DPPL-reinforced CTB.

The experimental design included both control and reinforced groups. The control specimens (designated N-3DPPL) with cement-to-tailings ratios of 1:4 (N-1:4), 1:6 (N-1:6), and 1:8 (N-1:8) contained no reinforcements. The reinforced groups featured three distinct 3DPPL configurations (four-column FC-1:8, six-column SC-1:8, and cross-shaped CO-1:8) at a fixed 1:8 cement-to-tailings ratio. All mixtures maintained 70% solid concentration, with three identical specimens prepared for each formulation to ensure statistical reliability (final strength values represent averaged results). Material preparation involved precise weighing (0.01 g accuracy) using an electronic balance, followed by 3 min mixing in a mortar mixer to achieve homogeneous slurry. The 3DPPL structures were positioned in standard molds prior to slurry pouring. Specimens underwent initial curing (24 h at 20 ± 1 °C and 95 ± 5% RH) before demolding, followed by 7-day curing under identical environmental conditions (automatically maintained). Figure 4 details the complete specimen preparation protocol.

### 2.3. Strength Test and DIC Analysis

**Figure 5:**
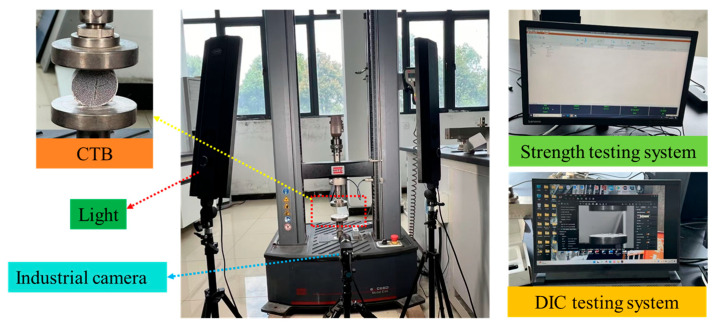
The pictures of the strength test and DIC analysis equipment.

Brazilian splitting tests were conducted to evaluate the tensile performance of the backfill materials, which experience significant tensile stresses during mine support and goaf stabilization [70]. Specimens were surface-polished to ensure parallel contact planes before testing. Using an Empyrean series MTS universal testing machine (30 kN capacity), we performed tests at constant loading rates while automatically recording the complete failure process and load–displacement curves until specimen failure. Each mixture formulation was tested in triplicate, with reported strength values representing averages. Digital Image Correlation (DIC) analysis monitored the strain distribution and fracture evolution using a speckle pattern (0.5 mm dots) captured by synchronized industrial cameras. The GOM Correlate 2019 software processed high-definition images by tracking speckle displacement to automatically calculate the strain fields in strain mode. Figure 5 illustrates the complete experimental setup for both the strength testing and DIC measurements.

### 2.4. Microstructure Analysis Was Conducted Through SEM

The scanning electron microscopy (SEM) analysis was conducted to examine the internal microstructure of CTB specimens, assessing their compactness and homogeneity [71]. Microstructural characterization was performed using a Zeiss Sigma 300 SEM (1.0 nm resolution, 30 kV operating voltage, 10–1,000,000× magnification range, Oberkochen, Germany). Prior to imaging, specimens were sputter-coated with gold to ensure proper conductivity and placed in the vacuum chamber for evacuation.

## 3. Results and Discussion

### 3.1. The Tensile Strength of All CTB

The stress–strain curves of the backfill specimens were obtained using a universal testing machine, from which the peak stress was determined and converted to tensile strength, as calculated through Formula (1) [72]:

(1) $$\sigma_{t}=\frac{2P}{\pi DH},$$

where *σ_t_* denotes the tensile strength of CTB (MPa), *P* represents the peak force (N), *D* indicates the diameter of the specimen’s cross section (mm), and *H* corresponds to the height of the specimen (mm), with all parameters being measured under standardized testing conditions to ensure data reliability.

**Figure 6:**
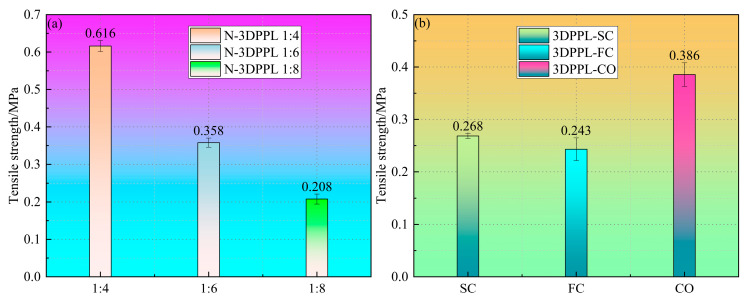
The tensile strength of CTB: (**a**) N-3DPPL reinforced CTB; (**b**) 3DPPL reinforced CTB.

Figure 6 shows the tensile strength results for all CTB specimens calculated using Equation (1). The control specimens (N-3DPPL) with cement-to-tailings ratios of 1:4, 1:6, and 1:8 showed decreasing tensile strengths (0.616, 0.358, and 0.208 MPa, respectively), confirming cement’s dominant role in strength development. At the 1:8 ratio, the 3DPPL-reinforced specimens exhibited significant strength improvements: four-column (FC) = 0.243 MPa, six-column (SC) = 0.268 MPa, and cross-shaped (CO) = 0.386 MPa. The CO configuration demonstrated superior performance, attributed to its biaxial stress transfer mechanism and physical constraints that restrict matrix deformation (the hardened continuous phase of tailings, cement and water). This cross-lattice structure enhanced synergistic deformation through complex interfacial geometry and mechanical interlocking, forcing cracks to propagate around multiple polymer interfaces. In comparison, the unidirectional columnar structures provided limited transverse restraint, resulting in weaker tensile strength enhancement.

To quantify the effect of 3DPPL on the CTB’s tensile strength, the strength growth rate (SGR) was calculated using Equation (2).

(2) $$SGR=\frac{S_{2}-S_{1}}{S_{1}}\times 100\%$$

where *S*_2_ denotes the tensile strength of 3DPPL-reinforced CTB, and *S*_1_ represents the tensile strength of the control group.

**Figure 7:**
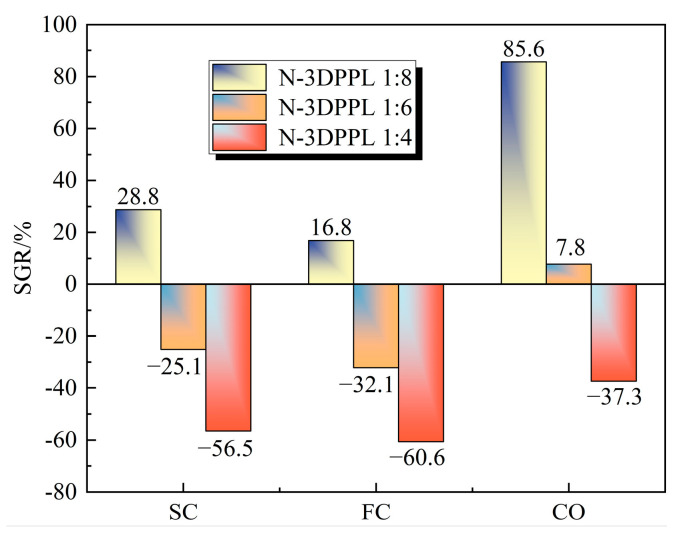
The SGR of 3DPPL-reinforced CTB.

The results of the SGR are shown in Figure 7. Compared to the control group, the 3DPPL-reinforced CTB with SC, FC, and CO structures exhibited enhanced strength. At a cement-to-tailings ratio of 1:8, the SGR values reached 28.8, 16.8, and 85.6%, respectively; at a 1:6 ratio, the SGR values were −25.1, −32.1, and 7.8%; while at a 1:4 ratio, they measured −56.5, −60.6, and −37.3%. These calculations demonstrated that 3DPPL effectively improved the CTB’s tensile strength. With reduced cement content, 3DPPL positively compensated for the tensile strength, as evidenced by the 7.8% strength increase in 1:6 and 1:8 ratio 3DPPL-reinforced specimens with a CO structure, which not only counteracted the negative effects of cement reduction but also provided additional strength enhancement.

### 3.2. The Load–Deflection Curves Obtained from the Brazilian Tensile Test

**Figure 8:**
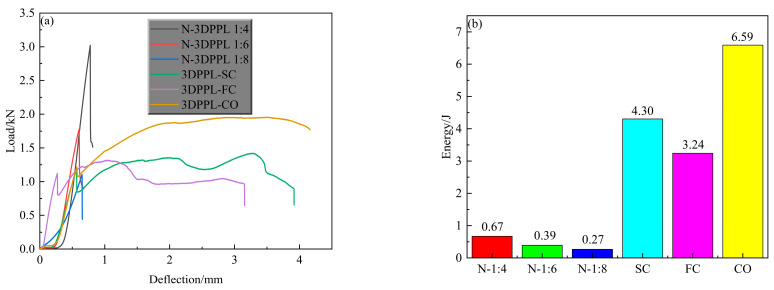
The load–displacement curve and energy of CTB: (**a**) load–displacement curve; (**b**) energy.

Figure 8a presents the tensile strength characteristics of CTB, revealing distinct curve patterns between the control specimens and 3DPPL-reinforced specimens. All control specimens exhibited brief ascending curves followed by rapid load reduction after peak load, indicating sudden failure. Typically, the load–deflection curves of CTB progress through four stages: pore compaction, linear elastic deformation, nonlinear elastic phase, and crack development [68]. While all specimens showed similar linear elastic behavior, the control specimens demonstrated abrupt post-peak load reduction with a negligible crack development phase, reflecting their pronounced brittle failure. In contrast, 3DPPL significantly prolonged the failure process of CTB, evidenced by greater deflection capacity and extended crack development phases with smoother transitional curves, demonstrating improved ductility and plastic failure characteristics. To quantify the ductility of CTB, the area enclosed by the curve and the *X*-axis was defined as the energy required for the complete failure of CTB, as shown in Figure 8b. The results show that 3DPPL significantly increased the energy required for the complete failure of CTB; that is, the plasticity of CTB is significantly improved. Especially for the CO structure, the energy required to completely disable CTB is 8.8 to 23.4 times that of ordinary CTB. The 3DPPL-reinforced specimens exhibited periodic load fluctuations during crack propagation, attributable to the sequential re-tensioning mechanism of 3DPPL structures: initial load-bearing by the outer matrix until its failure, subsequent stress transfer to 3DPPL, and progressive load redistribution until complete structural failure [73]. Notably, the CO-structured 3DPPL enabled higher maximum loads than the 1:6 ratio specimens, demonstrating partial cement substitution capability, though this effect remained limited compared to the 1:4 ratio specimens (exhibiting the highest load capacity). Yaya et al. [63] also found that 3DPPL changed the failure mode of CTB from brittle fracture to progressive failure, which was consistent with the DIC observation results of this study. Collectively, 3DPPL incorporation enhanced the peak load capacity, tensile strength, and ductility, with structural configuration significantly influencing the reinforcement efficacy.

### 3.3. The Failure Behavior of CTB Through DIC Analysis

**Figure 9:**
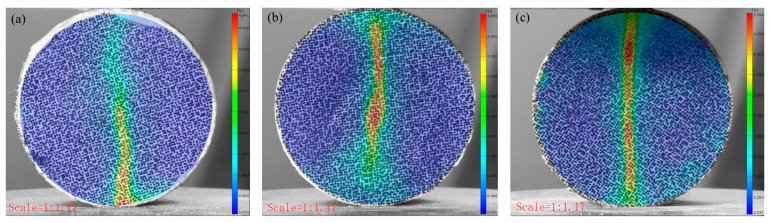
The damage situation of the control group specimens: (**a**) N-1:4; (**b**) N-1:6; (**c**) N-1:8.

**Figure 10:**
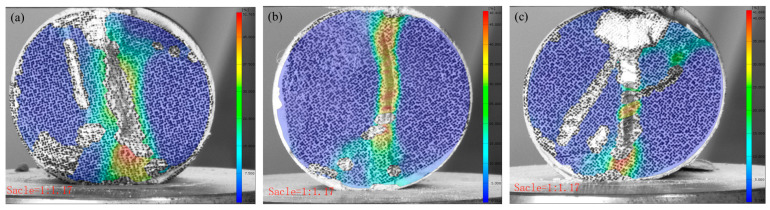
The damage situation of the 3DPPL-reinforced specimens: (**a**) CO; (**b**) SC; (**c**) FC.

Figure 9 and Figure 10 present the failure characteristics of the CTB specimens. The control specimens displayed typical tensile failure patterns, with vertical fractures propagating from the central region parallel to the loading direction. In contrast, the 3DPPL-reinforced specimens exhibited more complex failure modes, including widened primary cracks accompanied by secondary microcracks and shear-induced diagonal fractures near loading points. DIC analysis quantified these differences, revealing maximum strain values of 51.765, 48.41, and 41.698% at crack locations in reinforced specimens versus significantly lower values of 4.841, 4.05, and 2.504% in controls. This enhanced performance results from 3DPPL’s compensatory mechanism: while the reduced cement content increases the matrix brittleness, the lattice structure provides ductile behavior through interfacial bonding and stress redistribution [74]. During microcrack initiation, 3DPPL assumes partial tensile stresses, diverting crack propagation and creating oblique shear cracks. Unlike the control specimens that fractured cleanly into two halves, the reinforced specimens maintained structural integrity despite matrix spalling during progressive failure, though some fragment detachment occurred due to the lattice’s finite confinement capacity—consistent with observations by Liu et al. [75].

### 3.4. The Strain and Displacement Analysis of CTB Based on DIC Analysis

**Figure 11:**
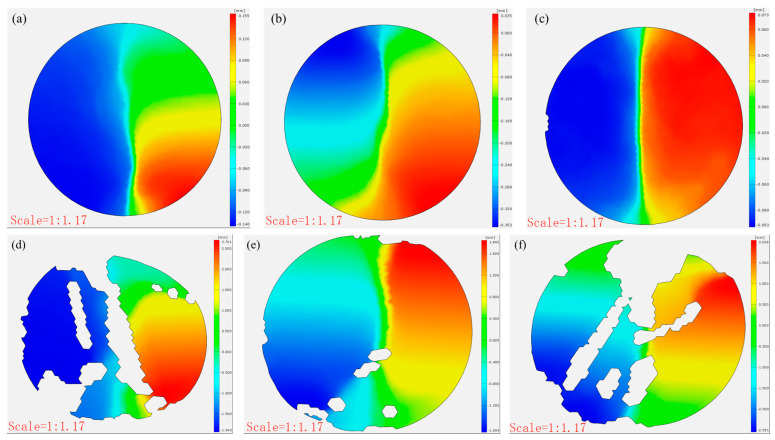
The horizontal displacement of CTB (**a**) N-1:4; (**b**) N-1:6; (**c**) N-1:8; (**d**) CO; (**e**) SC; (**f**) FC.

Figure 11 compares the post-failure horizontal displacements of the CTB specimens. The control specimens showed minimal x-direction displacement (0.155, 0.035, and 0.073 mm), demonstrating brittle failure with negligible deformation at peak load. In contrast, the 3DPPL-reinforced specimens exhibited substantially greater displacements (2.701, 1.842, and 2.038 mm), particularly in CO configurations, resulting from interfacial slippage at the 3DPPL–cement matrix weak zones during loading. This behavior reflects fundamentally different energy dissipation mechanisms: while the controls failed through brittle fracture alone, the reinforced specimens employed multiple mechanisms including interface debonding and polymer plastic deformation. These processes generated complex three-dimensional deformations, quantitatively captured through DIC measurements as increased horizontal displacements.

**Figure 12:**
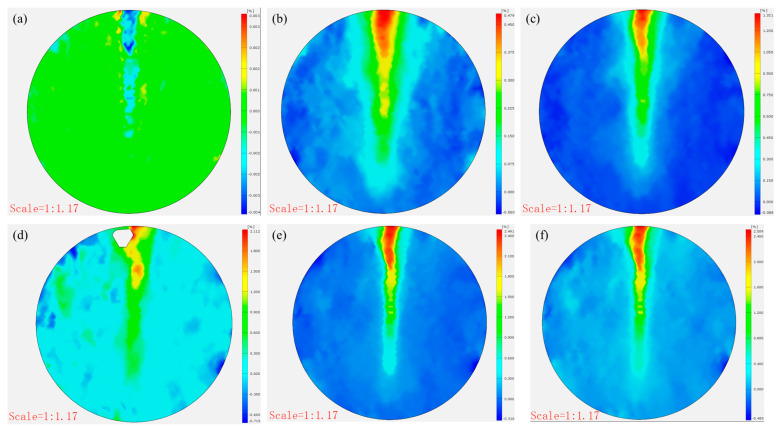
The strain evolution (**a**–**f**) in the x-direction of the control group specimens.

**Figure 13:**
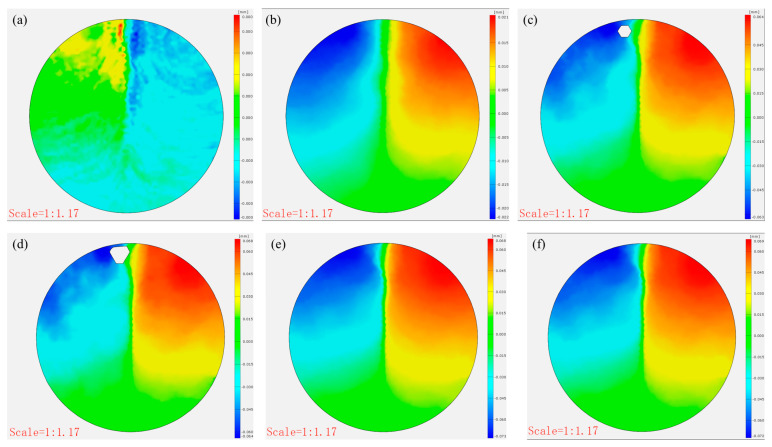
The horizontal displacement evolution (**a**–**f**) of the control group specimen.

**Figure 14:**
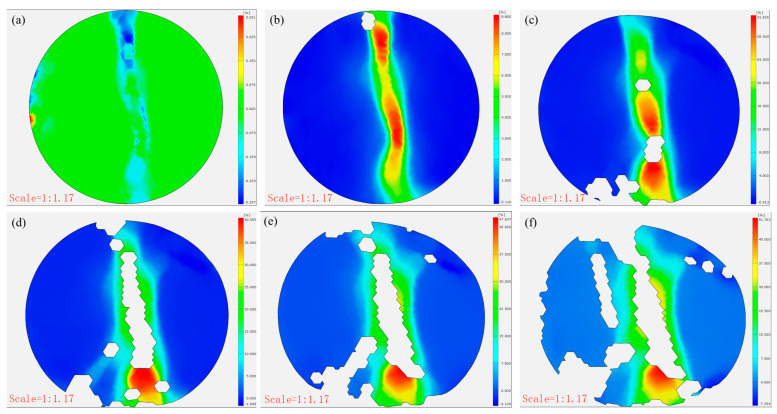
The strain evolution (**a**–**f**) in the x-direction of the CO-structured 3DPPL-reinforced CTB.

**Figure 15:**
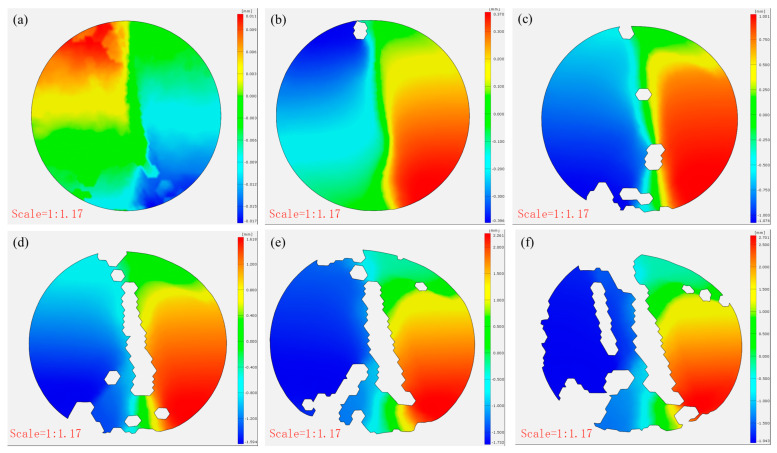
The horizontal displacement evolution (**a**–**f**) of the CO-structured 3DPPL-reinforced CTB.

Figure 12 and Figure 13 present the damage progression recorded via DIC testing for control specimens with a cement-to-tailings ratio of 1:8 at different loading stages. This reveals that the main crack reached a stable propagation phase without significant further development. At the same time, the strain gradually increased at a decelerating rate (0.479–1.321–2.112–2.491–2.509%), and the x-direction displacement incrementally rose (0.021 mm–0.064 mm–0.068 mm–0.069 mm), attributable to time-dependent creep mechanisms governing damage evolution after crack stabilization until final failure. In contrast, Figure 14 and Figure 15 demonstrate the damage progression in CO-structured 3DPPL-reinforced specimens, where rapid initial strain development (8.88–31.626%) accompanied main crack formation, followed by secondary crack propagation. The strain development similarly exhibited a gradual deceleration, with the maximum strain reaching 20.6 times that of the control specimens. Correspondingly, the displacement in the x-direction progressively increased, attaining a maximum value 39.1 times higher than that observed in the control specimens. These observations demonstrate that while 3DPPL enhanced the deformation capacity of CTB, it simultaneously accelerated primary crack initiation; however, the decelerating strain development rate indicated maintained load-bearing capacity during crack propagation, exhibiting distinct ductile characteristics. Collectively, 3DPPL incorporation significantly improved both the crack resistance and toughness of CTB.

### 3.5. The Microscopic Analysis Through SEM

**Figure 16:**
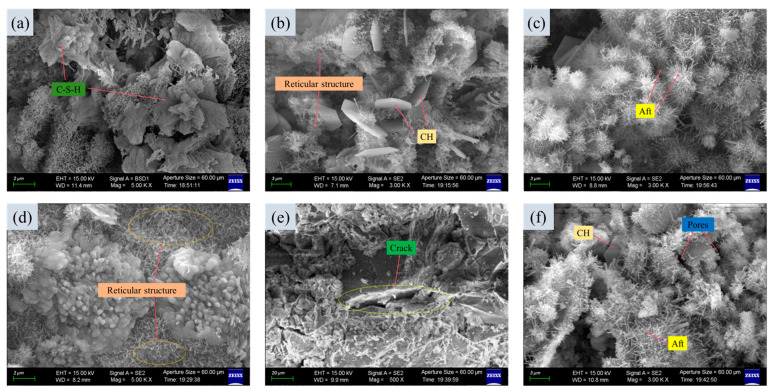
The microscopic structure diagram (**a**–**f**) of the CTB matrix.

SEM analysis of the 3DPPL-reinforced specimens’ microstructure revealed its enhancement mechanism. Figure 16 distinctly shows the following: C-S-H gel forming the primary binding phase that connects tailings particles, needle-like AFt crystals mechanically interlocking with surface pores of the 3DPPL, and CH accumulating at the polymer–cement interface [76]. These hydration products are respectively shown in Figure 16a–c, which form a stable network structure as illustrated in Figure 16d. However, the inherent pores within the backfill material were observed to adversely affect its mechanical strength [77].

There was crack deflection along the 3DPPL–matrix interface, rather than penetrating the polymer lattice, and controlled interfacial debonding that absorbed fracture energy. This microstructural evidence explained the macroscopic performance, where the 3DPPL establishes a multi-scale reinforcement system—with SC/FC configurations mitigating tensile stresses through axial load-bearing, while the CO structures suppressed cracks via spatial truss effects. Additionally, the 3DPPL enhanced the toughness through energy dissipation mechanisms involving polymer fiber bridging that consumed crack propagation energy, lattice-matrix interfacial debonding that absorbed fracture energy, and interlocking structures from oriented hydration products that strengthened interfacial bonding. This multi-scale synergy transforms the CTB’s failure mode from brittle fracture to progressive damage accumulation, significantly improving the tensile strength and ductility. The hydrophobic organic–inorganic interface effectively redistributes stress through the hybrid layer, combining polymer fiber bridging that consumes crack energy with interfacial debonding that enhances toughness. 3DPPL transformed CTB’s failure mode from brittle fracture to progressive damage accumulation, as the 3DPPL provided continuous energy absorption until either interfacial delamination or lattice fracture occurred. Meanwhile, 3DPPL provided continuous energy absorption through deformation until either interfacial delamination with the CTB matrix or lattice fracture occurred. With increasing load application, cracks progressively developed within the CTB until final failure occurred, as demonstrated in Figure 16e.

**Figure 17:**
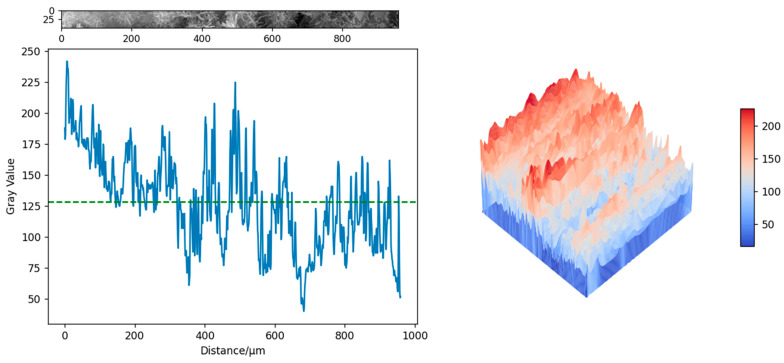
The gray value of the control group specimen.

**Figure 18:**
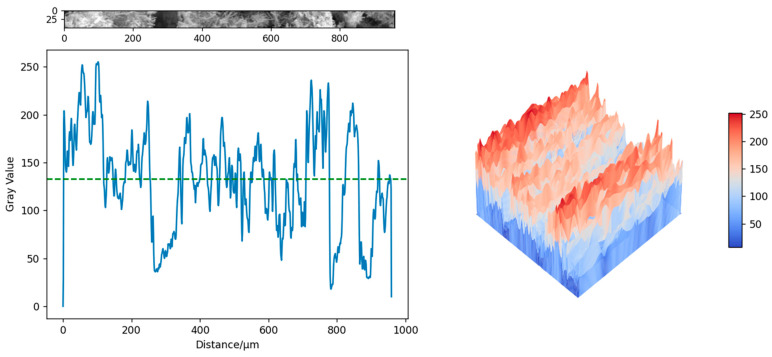
The gray value of the 3DPPL-reinforced specimen.

The gray value analysis was conducted on the specimens shown in Figure 16c,f, which were, respectively, the test specimens of the control group and the 3DPPL-enhanced specimens with the CO structure. The grayscale values are converted into three-dimensional images through interpolation functions in NumPY 1.26.4, Matplotlib 3.8.4 and SciPy 1.12.0 [78], as shown in Figure 17 and Figure 18. The principle was that in SEM testing, the denser the structure, the smaller the interaction between the electron beam and the interior of the specimen and the larger the gray value [79]. The results showed that the gray values of the blank group specimen and 3DPPL-reinforced specimen were 128.4 and 133.2, respectively. This result indicated that 3DPPL and the CTB matrix can be well combined. Because the gray value of the specimen was larger and there were fewer pores, its density was better. This was because the 3D-printed polymer lattice, as a reinforcing framework, was embedded in the mortar system, which guided the directional deposition of cement hydration products and reduced the formation of disordered pores.

## 4. Conclusions

This study investigated the strength characteristics of 3DPPL-reinforced CTB with three distinct 3DPPL configurations (four-column, six-column, and cross-shaped structures) through Brazilian splitting tests, with the significant effects of 3DPPL validated via tensile strength test and load–deflection curve analysis. The crack damage behavior and strain distribution under 3DPPL reinforcement were characterized using DIC analysis, while SEM analysis elucidated the underlying reinforcement mechanisms. The main conclusions are as follows:

(1) The 3DPPL significantly enhanced the tensile performance of CTB, with the CO structure demonstrating the most pronounced reinforcement effect, achieving an 85.6% increase in tensile strength, reaching 0.386 MPa at a cement-to-tailings ratio of 1:8, exhibiting considerable potential as a cement replacement material.

(2) The 3DPPL reinforcement endowed the CTB with significantly improved ductility, contrasting sharply with the brittle failure characteristics of the control specimens. The load–deflection curves of the 3DPPL-reinforced specimens exhibited distinct multi-stage characteristics featuring prolonged crack development phases and periodic fluctuations.

(3) The 3DPPL reinforcement induced a combined tensile-shear failure mode in CTB, characterized by multi-crack propagation patterns. All 3DPPL-reinforced specimens exhibited substantially higher strain concentrations at fracture locations, overcoming the brittleness inherent while significantly enhancing the CTB’s ductility.

(4) The CO-structured specimens demonstrated superior deformation capacity with a maximum horizontal strain of 51.763% and an x-direction displacement of 2.701 mm, representing 20.6- and 39.1-times increases compared with control specimens, respectively, improving the crack resistance and toughness of CTB.

(5) The 3DPPL transformed the failure mode of CTB from brittle fracture to progressive failure, achieving a synergistic enhancement of both strength and toughness.

Future research should explore practical implementation strategies for 3DPPL-reinforced CTB in real-world mining applications, focusing on scalable fabrication and deployment methods. For large-scale terrain remediation, we envision a two-phase implementation approach: (a) prefabrication of modular 3DPPL units using industrial-scale 3D printers, with interlocking geometries allowing on-site assembly like building blocks, and (b) in-situ printing of lattice structures directly in stopes using robotic arms mounted on mining equipment, where polymer resin would be extruded following pre-programmed patterns based on stope geometry scans. The technique’s viability hinges on developing mobile printing systems that can operate in underground environments, potentially using quick-curing polymers compatible with continuous backfill operations. The key challenges include ensuring print quality in dusty conditions, optimizing pumping systems for dual-material (polymer lattice + CTB slurry) deposition, and establishing quality control protocols for field conditions. Economic feasibility studies should compare the long-term benefits of enhanced stability against the capital costs of 3D printing infrastructure, while environmental assessments must verify the sustainability of large-scale polymer use in mining contexts. Initial pilot testing could begin with surface remediation projects before progressing to underground applications, allowing gradual optimization of the technology for different geological conditions and mining methods.

## 5. The Advantage of 3DPPL Application

To address the environmental and economic feasibility of 3DPPL in mining applications, our study demonstrates several key advantages: the cross-shaped 3DPPL configuration enables 50% cement reduction while maintaining mechanical performance, offering significant cost savings given cement typically accounts for 60–70% of backfill material costs. For field implementation, 3DPPL units are mass-produced off-site then assembled underground, this approach reduces on-site labor compared to traditional reinforcement methods. The technology could reduce CO_2_ emissions when considering cement reduction and transportation optimization. These findings suggest 3DPPL is both technically and economically viable for scale-up, particularly in large-scale operations where the higher initial polymer costs would be off-set by long-term savings from reduced cement use and improved stope stability.

## Figures and Tables

**Table 1:** The basic parameters of 3DPPL.

Polymer Material	Material Harness	Breaking Strength	Bending Strength	Bending Modulus	Tensile Strength	Heat Distortion Temperature
WR	87	70 MPa	84 MPa	2.8 GPa	60 MPa	46 °C

## Data Availability

The original contributions presented in this study are included in the article. Further inquiries can be directed to the corresponding authors.

## References

[1] Qiu J., Xiang J., Zhang W., Zhao Y., Sun X., Gu X. (2022). Effect of microbial-cemented on mechanical properties of iron tailings backfill and its mechanism analysis. Constr. Build. Mater..

[2] Khalil A., Ait-Khouia Y., Beniddar H., EI Ghorfi M. (2025). Sustainable reprocessing of Pb–Zn mine tailings through froth flotation for resource recovery and environmental remediation in abandoned mining regions. Miner. Eng..

[3] Meng X., Tang Z., Gao P., Zhang Y. (2025). Mechanistic Analysis of Hydrogen Mineral Phase-Transformed Iron Ore Tailings in Cementitious Materials: A Study on Hydration Kinetics, Mechanical Properties, and Microstructural Characteristics. Constr. Build. Mater..

[4] Jin J., Chen Y., Li M., Liu T., Qin Z., Liu Q., Liang B., Zhao J., Zuo S. (2024). Preparation of self-consolidating cemented backfill with tailings and alkali activated slurry: Performance evaluation and environmental impact. Constr. Build. Mater..

[5] Sun X., Xiang J., Xiong B., Kong X., Qiu J. (2024). Combined biological and cement solidification of lead-zinc tailings for backfill preparation and its environmental effects. Constr. Build. Mater..

[6] Zhang X., Xue X., Ding D., Sun P., Li J., He Y. (2024). A study of the mechanical properties, environmental effect, and microscopic mechanism of phosphorus slag-based uranium tailings backfilling materials. J. Clean. Prod..

[7] Benkirane O., Haruna S., Fall M. (2023). Strength and microstructure of cemented paste backfill modified with nano-silica particles and cured under non-isothermal conditions. Powder Technol..

[8] Wu J., Wong H.S., Yin Q., Ma D. (2023). Effects of aggregate strength and mass fraction on mesoscopic fracture characteristics of cemented rockfill from gangue as recycled aggregate. Compos. Struct..

[9] Liu Z., Gan D., Sun H., Xue Z., Zhang Y. (2025). Dynamic impact performance of cemented tailings backfill in a water-bearing environment: Coupling effects and damage characteristics. Soil Dyn. Earthq. Eng..

[10] Chen Q., Sun S., Liu Y., Qi C., Zhou H., Zhang Q. (2021). Immobilization and leaching characteristics of fluoride from phosphogypsum-based cemented paste backfill. Int. J. Miner. Metall. Mater..

[11] Chen W., Chen L., Yin S. (2024). Effect of bacteria-fly-ash based binder on cemented tailings backfill: Mechanical strength, solidified mechanism and economic benefits. Constr. Build. Mater..

[12] Wu D., Zhao P., Cheng W., Hao Z., Zhang Y. (2024). Effect of microwave on coupled rheological and mechanical properties of cemented tailings backfill. Constr. Build. Mater..

[13] Zhang S., Chen S., Min L., Yin L., Rui H., Zhang H. (2024). Study on the evolution of mechanical properties of backfill body under the combined action of sulfate erosion and load. Case Stud. Constr. Mater..

[14] Zhao B., Wen J., Zhai D., Tang R., Chen S., Xin J. (2025). Effect of calcium chloride on the properties of gangue cemented paste backfill: Experimental results of setting time, rheological properties, mechanical strength and microscopic properties. Case Stud. Constr. Mater..

[15] Al-Moselly Z., Fall M. (2024). Multiphysical testing of strength development of cemented paste backfill containing superplasticizer. Cem. Concr. Compos..

[16] Haruna S., Fall M. (2021). Strength development of cemented tailings materials containing polycarboxylate ether-based superplasticizer: Experimental results on the effect of time and temperature. Can. J. Civ. Eng..

[17] Zou S., Gao Y., Zhou Y., Sun H., Yang Z., Yang C., Jiang H. (2025). Study on the multi-scale tensile mechanical behavior of fiber reinforced cemented tailings backfill considering surface roughness effects. Constr. Build. Mater..

[18] Huang Z., Cao S., Yilmaz E. (2021). Investigation on the flexural strength, failure pattern and microstructural characteristics of combined fibers reinforced cemented tailings backfill. Constr. Build. Mater..

[19] Zhao K., Shi Y., Yan Y., Ma C., Liu Y., Nie Q. (2025). Acoustic emission response precursor characterization of cemented tailings backfill at different loading rates. Constr. Build. Mater..

[20] Yang L., Kou Y., Li G., Chen M., Zhu G., Song Z., Wang P. (2024). Investigation of macro-micro mechanical behaviors and failure mechanisms in cemented tailings backfill with varying proportions of fine. Constr. Build. Mater..

[21] Li J., Yilmaz E., Cao S. (2020). Influence of solid content, cement/tailings ratio, and curing time on rheology and strength of cemented tailings backfill. Minerals.

[22] Wang Z., Wang Y., Cui L., Bi C., Wu A. (2022). Insight into the isothermal multiphysics processes in cemented paste backfill: Effect of curing time and cement-to-tailings ratio. Constr. Build. Mater..

[23] Libos I.L.S., Cui L. (2020). Effects of curing time, cement content, and saturation state on mode-I fracture toughness of cemented paste backfill. Eng. Fract. Mech..

[24] Pan H., Xiao Q., Huang N., Xiao A., Zhu D. (2024). Effect of alkaline rice straw fibers content and curing ages on static mechanical properties of cemented lithium mica tailings backfill. Case Stud. Constr. Mater..

[25] Xin J., Liu L., Xu L., Wang J., Qu H. (2022). A preliminary study of aeolian sand-cement-modified gasification slag-paste backfill: Fluidity, microstructure, and leaching risks. Sci. Total Environ..

[26] Jin J., Liu T., Li M., Lv T., Zhang S., Zuo S. (2025). Mechanical properties and compression damage characteristics of coal gangue-filled backfill cemented by fly ash modified self-consolidating grouts. Case Stud. Constr. Mater..

[27] Zhu D., Huang N., Li W., Li J., Wu X. (2024). Effect of different fibers and fiber contents on the mechanical properties and failure behavior of early age cemented lithium feldspar tailings backfill. Dev. Built Environ..

[28] Zou S., Guo W., Wang S., Gao Y., Qian L., Zhou Y. (2023). Investigation of the dynamic mechanical properties and damage mechanisms of fiber-reinforced cemented tailing backfill under triaxial split-Hopkinson pressure bar testing. J. Mater. Res. Technol..

[29] Hu Y., Zhang B., Zhu S., Han B., Zheng L., Chen D., Liu Z. (2025). Preparation of high-performance and environmentally friendly superfine tailings cemented paste backfill using cellulose nanofibers. Process Saf. Environ. Prot..

[30] Zhu T., Chen Z., Wang Z., Cao J., Hao J., Zhou Z. (2025). Energy damage evolution and mesoscopic failure mechanism of cemented waste rock tailing backfill under axial compression. Structures.

[31] Huang J., Wu C., Huang N., Deng L., Zhu D. (2025). Utilization of waste rock from a low-carbon perspective: Mechanical performance analysis of waste rock-cemented tailings backfill. J. CO2 Util..

[32] Chen X., Shi X., Zhou J., Yu Z., Huang P. (2020). Determination of mechanical, flowability, and microstructural properties of cemented tailings backfill containing rice straw. Constr. Build. Mater..

[33] Liu W., Yu H., Wang S., Wei M., Wang X., Tao T., Song X. (2023). Evolution mechanism of mechanical properties of cemented tailings backfill with partial replacement of cement by rice straw ash at different binder content. Powder Technol..

[34] Guo Z., Qiu J., Kirichek A., Zhou H., Liu C., Yang L. (2024). Recycling waste tyre polymer for production of fibre reinforced cemented tailings backfill in green mining. Sci. Total Environ..

[35] Su L., Wu S., Yang J., Zhang M., Zhu W., Fu X. (2025). Experimental study on the dynamic mechanical properties of polypropylene fibre-reinforced coal gangue-cemented backfill. Constr. Build. Mater..

[36] Zou S., Gao Y., Zhou Y., Sun H., Yang Z., Yang C., Chai J., Qian L. (2025). Mechanical Properties of Organic-Inorganic Hybrid Fiber Reinforced Cemented Tailings Backfill Considering Energy Evolution and Damage Fracture Characteristics. J. Mater. Res. Technol..

[37] Yin S., Hou Y., Chen X., Zhang M., Du H., Gao C. (2022). Mechanical behavior, failure pattern and damage evolution of fiber-reinforced cemented sulfur tailings backfill under uniaxial loading. Constr. Build. Mater..

[38] Wang J., Yu Q., Xiang Z., Fu J., Wang L., Song W. (2023). Influence of basalt fiber on pore structure, mechanical performance and damage evolution of cemented tailings backfill. J. Mater. Res. Technol..

[39] Hou Y., Yang K., Yin S., Yu X., Kou P., Wang Y. (2024). Enhancing workability, strength, and microstructure of cemented tailings backfill through mineral admixtures and fibers. J. Build. Eng..

[40] Xue G., Yilmaz E. (2022). Strength, acoustic, and fractal behavior of fiber reinforced cemented tailings backfill subjected to triaxial compression loads. Constr. Build. Mater..

[41] Li Z., Zhang S., Wu C., Zhu D. (2024). Influence of fiber type and content on the flexural strength of cemented lithium feldspar tailings backfill composites. J. CO2 Util..

[42] Zhang S., Zhao T., Li Y., Li Z., Li H., Zhang B., Li J., Li Y., Ni W. (2024). The effects and solidification characteristics of municipal solid waste incineration fly ash-slag-tailing based backfill blocks in underground mine condition. Constr. Build. Mater..

[43] Wang A., Cao S., Yilmaz E. (2023). Quantitative analysis of pore characteristics of nanocellulose reinforced cementitious tailings fills using 3D reconstruction of CT images. J. Mater. Res. Technol..

[44] Zhu T., Chen Z., Cao J., Wang Z., Hao J., Zhou Z. (2025). Crack resistance of cemented waste rock tailings backfill under splitting tensile load: Experimental and numerical investigations. J. Build. Eng..

[45] Zhang L., Lai X., Pan J., Shan P., Zhang Y., Zhang Y., Xu H., Cai M., Xi X. (2024). Experimental investigation on the mixture optimization and failure mechanism of cemented backfill with coal gangue and fly ash. Powder Technol..

[46] Jiang X., Lang L., Liu S., Mu F., Wang Y., Zhang Z., Han L., Duan S., Wang P., Li J. (2024). Stabilization of iron ore tailing with low-carbon lime/carbide slag-activated ground granulated blast-furnace slag and coal fly ash. Constr. Build. Mater..

[47] Cheng Y., Shen H., Zhang J. (2023). Understanding the effect of high-volume fly ash on micro-structure and mechanical properties of cemented coal gangue paste backfill. Constr. Build. Mater..

[48] Wang A., Cao S., Yilmaz E. (2022). Influence of types and contents of nano cellulose materials as reinforcement on stability performance of cementitious tailings backfill. Constr. Build. Mater..

[49] Cao S., Che C., Zhang Y., Shan C., Liu Y., Zhao C., Du S. (2024). Mechanical properties and damage evolution characteristics of waste tire steel fiber-modified cemented paste backfill. Int. J. Min. Sci. Technol..

[50] Zhu T., Chen Z., Wang Z., Cao J., Hao J., Zhou Z. (2025). Experimental and DEM analyses of type I fracture characteristics of waste rock aggregate reinforced cemented tailing backfill. Theor. Appl. Fract. Mech..

[51] Wang B., Kang M., Liu C., Yang L., Li Q., Zhou S. (2024). Experimental study on mechanical and microstructure properties of cemented tailings-waste rock backfill with continuous gradation. J. Build. Eng..

[52] Wu J., Jing H., Yin Q., Meng B., Han G. (2020). Strength and ultrasonic properties of cemented waste rock backfill considering confining pressure, dosage and particle size effects. Constr. Build. Mater..

[53] Takva Ç., Top S.M., Gökgöz B.İ., Gebel Ş., Ilerisoy Z.Y., Ilcan H., Şahmaran M. (2024). Applicability of 3D concrete printing technology in building construction with different architectural design decisions in housing. J. Build. Eng..

[54] Wu H.J., Diao J.B., Li X.R., Yue D.M., He G.H., Jiang X.B., Li P.P. (2025). Hydrogel-based 3D printing technology: From interfacial engineering to precision medicine. Adv. Colloid Interface Sci..

[55] Shahpasand R., Talebian A., Mishra S.S. (2023). Investigating environmental and economic impacts of the 3D printing technology on supply chains: The case of tire production. J. Clean. Prod..

[56] Chakraborty S., Biswas M.C. (2020). 3D printing technology of polymer-fiber composites in textile and fashion industry: A potential roadmap of concept to consumer. Compos. Struct..

[57] Fang R.C., Wang B.X., Pan J.J., Liu J.Q., Wang Z.H., Wang Q., Ling X.Z. (2023). Effect of concrete surface roughness on shear strength of frozen soil–concrete interface based on 3D printing technology. Constr. Build. Mater..

[58] Kantaros A., Piromalis D. (2021). Fabricating lattice structures via 3D printing: The case of porous bio-engineered scaffolds. Appl. Mech..

[59] Silva R.G., Estay C.S., Pavez G.M., Viñuela J.Z., Torres M.J. (2021). Influence of geometric and manufacturing parameters on the compressive behavior of 3D printed polymer lattice structures. Materials.

[60] Kantaros A. (2022). 3D printing in regenerative medicine: Technologies and resources utilized. Int. J. Mol. Sci..

[61] Zou S., Cao S., Yilmaz E. (2024). Enhancing flexural property and mesoscopic mechanism of cementitious tailings backfill fabricated with 3D-printed polymers. Constr. Build. Mater..

[62] Yaya N.S., Cao S., Yilmaz E. (2024). Effect of 3D printed skeleton shapes on strength behavior, stress evolution and microstructural response of cement-based tailings backfills. Constr. Build. Mater..

[63] Zhang H., Cao S., Yilmaz E. (2023). Polymer shape effect on damage evolution, internal defects and crack propagation of 3D-printed polymer-based cementitious backfill. Constr. Build. Mater..

[64] Li J., Cao S., Song W. (2023). Flexural behavior of cementitious backfill composites reinforced by various 3D printed polymeric lattices. Compos. Struct..

[65] Qin S., Cao S., Yilmaz E. (2022). Employing U-shaped 3D printed polymer to improve flexural properties of cementitious tailings backfills. Constr. Build. Mater..

[66] Qin S., Cao S., Yilmaz E., Li J. (2021). Influence of types and shapes of 3D printed polymeric lattice on ductility performance of cementitious backfill composites. Constr. Build. Mater..

[67] Xue G., Yilmaz E., Song W., Yilmaz E. (2019). Influence of fiber reinforcement on mechanical behavior and microstructural properties of cemented tailings backfill. Constr. Build. Mater..

[68] Grice T. Underground mining with backfill. Proceedings of the 2nd Annual Summit œ Mine Tailings Disposal Systems.

[69] Hu L., Zheng B., Zhu D., Yang Z., Huang N. (2025). Enhancing bending performance of cemented lithium feldspar tailings backfill with 3D printing polymer lattices: Effects of unit shapes and materials. Case Stud. Constr. Mater..

[70] Gao Y., Yu Z., Cheng Z., Chen W., Zhang T., Wu J. (2023). Influence of industrial graphene oxide on tensile behavior of cemented waste rock backfill. Constr. Build. Mater..

[71] Zhang L., Guo L., Liu S., Wei X., Zhao Y., Li M. (2024). A comparative study on the workability, mechanical properties and microstructure of cemented fine tailings backfill with different binder. Constr. Build. Mater..

[72] Zhang N., Hedayat A., Sosa H.G.B., Bernal R.P.H., Tupa N., Morales I.Y., Loza R.S.C. (2021). On the incorporation of class F fly-ash to enhance the geopolymerization effects and splitting tensile strength of the gold mine tailings-based geopolymer. Constr. Build. Mater..

[73] Liu B., Chen Y., Li D., Wang Y., Geng S., Qian K. (2024). Study on the fracture behavior and anisotropy of 3D-printing PVA fiber-reinforced concrete. Constr. Build. Mater..

[74] Vemuganti S., Soliman E., Taha M.M.R. Manipulating interfacial bond for controlling load transfer in 3D printed fiber reinforced polymer composites. J. Reinf. Plast. Compos..

[75] Liu Y., Zhang Z., Shi C., Zhu D., Li N., Deng Y. (2020). Development of ultra-high performance geopolymer concrete (UHPGC): Influence of steel fiber on mechanical properties. Cem. Concr. Compos..

[76] Wang T., Wu K., Wu M. (2020). Development of green binder systems based on flue gas desulfurization gypsum and fly ash incorporating slag or steel slag powders. Constr. Build. Mater..

[77] Zhu W., Yang L., Hou C., Zhu G., Liu X., Liang P. (2025). Effect of chloride salts on shear behavior of cemented tailings backfill (CTB) mixed with mine water in coastal regions. Int. J. Rock Mech. Min. Sci..

[78] Zuo L., Huang N., Wang G., Zhu D. (2024). Influence of polypropylene fiber length and geometric shape on the compressive strength of cemented lepidolite tailings backfill. Front. Mater..

[79] Mendoza F., Lu R. (2015). Basics of image analysis. Hyperspectral Imaging Technology in Food and Agriculture.

